# Neuropsychiatric Burden in Huntington’s Disease

**DOI:** 10.3390/brainsci7060067

**Published:** 2017-06-16

**Authors:** Ricardo Augusto Paoli, Andrea Botturi, Andrea Ciammola, Vincenzo Silani, Cecilia Prunas, Claudio Lucchiari, Elisa Zugno, Elisabetta Caletti

**Affiliations:** 1 Department of Psychiatry, Università degli Studi di Milano, Fondazione IRCCS Ca’ Granda Ospedale Maggiore Policlinico, Milan 20122, Italy; riccardo.paoli@guest.unimi.it (R.A.P.); crprunas@gmail.com (C.P.); elisazugno@gmail.com (E.Z.); calettielisabetta@gmail.com (E.C.); 2 Department of Neuro-Oncology, Fondazione IRCCS Istituto Neurologico Carlo Besta, Milan 20133, Italy; 3 Department of Neurology-Stroke Unit and Laboratory of Neuroscience, IRCCS Istituto Auxologico Italiano, Milan 20149, Italy; a.ciammola@auxologico.it (A.C.); vincenzo@silani.com (V.S.); 4 Department of Pathophysiology and Transplantation, “Dino Ferrari” Center, Università degli Studi di Milano, Milan 20122, Italy; 5 Department of Philosophy, Università degli Studi di Milano, Milan 20122, Italy; claudio.lucchiari@unimi.it

**Keywords:** Huntington’s disease, psychiatric symptoms, cognitive impairment, affective and nonaffective disorders.

## Abstract

Huntington’s disease is a disorder that results in motor, cognitive, and psychiatric problems. The symptoms often take different forms and the presence of disturbances of the psychic sphere reduces patients’ autonomy and quality of life, also impacting patients’ social life. It is estimated that a prevalence between 33% and 76% of the main psychiatric syndromes may arise in different phases of the disease, often in atypical form, even 20 years before the onset of chorea and dementia. We present a narrative review of the literature describing the main psychopathological patterns that may be found in Huntington’s disease, searching for a related article in the main database sources (Medline, ISI Web of Knowledge, Scopus, and Medscape). Psychiatric conditions were classified into two main categories: affective and nonaffective disorders/symptoms; and anxiety and neuropsychiatric features such as apathy and irritability. Though the literature is extensive, it is not always convergent, probably due to the high heterogeneity of methods used. We summarize main papers for pathology and sample size, in order to present a synoptic vision of the argument. Since the association between Huntington’s disease and psychiatric symptoms was demonstrated, we argue that the prevalent and more invalidating psychiatric components should be recognized as early as possible during the disease course in order to best address psychopharmacological therapy, improve quality of life, and also reduce burden on caregivers.

## 1. Introduction

Huntington’s disease (HD) is a neurodegenerative disease inherited in an autosomal dominant fashion, characterized by progressive movement disorders associated with cognitive and psychiatric symptoms [1,2]. The first clinical picture of the illness was provided in 1842 by Waters. Not until 1872 did George Huntington name the disease Huntington’s chorea, after a complete description of the symptoms and clinical course. By the 1980s, the name of the disease became known as Huntington’s disease, with the recognition of its motor and non-motor signs and symptoms [3]. Following the introduction of molecular biology techniques, a linkage was found on chromosome 4 in 1983, and 10 years later the gene for HD was discovered [2]. The underlying genetic defect is an unstable CAG trinucleotide repeat expansion in exon 1 of the HD gene, formerly called IT-15, on the short arm of chromosome 4. A repeat CAG length of 36 and longer is pathogenic and results in the synthesis of an abnormal polyglutamic tract in Huntington’s, a widely expressed protein of uncertain function [4,5] causing accumulation of intracellular protein aggregates, neurotrophic factor deprivation, impairment of energetic metabolism, transcriptional deregulation and, finally, hyperactivation of programmed cell-death mechanisms. Progressive dysfunction and neuronal loss, mainly in the caudate nucleus and in the putamen, start several years before the onset of motor symptoms. Along with basal ganglia pathology, neurodegeneration occurs in a large cortical region, in the thalamus, hypothalamus, and cerebellum [6,7].

Dopamine, glutamate, and γ-aminobutyric acid are thought to be the most affected neurotransmitters in HD and are currently the focus of pharmacotherapy [8]. The age of HD onset is inversely correlated with the length of the expansion in people with 40 or more CAG repeats, who will inevitably manifest the disease if they live a normal lifespan. In fact, when a number of CAG repeats between 36 and 39 presents, the penetrance is variable. In addition, it has been suggested that there may be subtle abnormalities, possibly constituting an endophenotype, in the rare individuals who have repeat lengths in the 27–35 range [1,2,6]. A recent systematic review by Wexler et al. [9] estimated a general incidence rate of 7.2 million people/years.

A meta-regression made by Pringssheim et al. [10] confirmed a significantly lower prevalence of HD in Asia compared to Europe, North America, and Australia. In particular, recent studies in a different part of the world reported that the prevalence should be updated with respect to older studies before the advent of the molecular diagnosis of Huntington disease (HTT) studies. For instance, Fisher et al [11] reported a prevalence in Canada of 17.2 in the Caucasian population per 100.00 and of 13.7 per 100.000 in the general population, while an Italian study reported a prevalence of 10.85 per 100.000 [12]. A lower prevalence is registered in Japan [13,14].

In most cases, the age of onset of HD is between 35 and 45 years, whereas the mean duration of the disease is 16 years [15,16]. Different stages of the disease may be described (premanifest, with soft signs, phenoconversion, and manifest), each being characterized by decreasing independence and the need for help caused by a deterioration of motor and cognitive performance and the presence of psychiatric symptoms [13].

Currently, symptomatic treatment is available for motor and psychiatric symptoms, but there is still no evidence that therapy can slow the evolution of brain degeneration.

## 2. Methods

Searches were conducted through Medline, ISI Web of Knowledge, Scopus, and Medscape. The following keywords or combinations were used: “apathy”, “behavioral”, “depression”, “irritability”, “neuropsychiatric symptoms”, “psychiatric”, “psychotic”, “sexuality”, and “suicidal”. We did not consider other terms that might impact psychiatric manifestations such as sleep, aggression, and impulsive, in order to focus the search on the main psychiatric symptoms. In so doing, some studies about specific psychiatric manifestations might have been excluded.

A total of 3055 papers were initially identified. A screening of the articles’ abstracts was then performed in order to consider only those articles that concerned with the topic of the review and removing duplicates.

Additional papers were identified during the reading (also during the revision process) and analyzed to be assessed as a potential data source.

The initial search identified 3055 articles. One thousand eight hundred and twenty-nine papers matched with term “psychiatric”, 495 “depression”, 137 “psychotic”, 43 “suicidal”, 35 “sexuality”, 62 “apathy”, 245 “behavioral”, 135 “neuropsychiatric symptoms”, and 74 “irritability”. An initial review of the titles and abstracts of these articles by three of the authors identified 238 articles that were potentially relevant to the current review. The abstracts of these articles were then evaluated against the inclusion criteria by the authors, resulting in 82 articles being identified as eligible for inclusion, with an additional six articles identified during manuscript preparation, for a total of 88 articles. This review provides a narrative synthesis of the findings from previous key reviews and empirical studies identified in the literature search.

Inclusion and exclusion criteria:

We included articles assessing the prevalence, incidence, and phenomenology of neuropsychiatric and psychiatric symptoms in patients with HD. We included only articles reporting data about patients with a genetic diagnosis. This suggested a lower time limit to the search that started from 1983. We also included articles reporting data about the preclinical presentation of psychiatric and neuropsychiatric symptoms. We didn’t posit limits to the sample size of the studies considered.

We excluded reviews, editorials, opinion papers, letters, surveys, and articles not clearly reporting the methodology used, the sample size, and/or not written in English.

## 3. Clinical Features in HD and Psychiatric Disorders

Clinical pictures of HD comprise motor abnormalities (chorea, dystonia, bradykinesia, oculomotor dysfunction), cognitive impairment, behavioral problems, and psychiatric disorders. The latter are major constituents of the clinical spectrum of HD and have a substantial impact on daily functioning, constituting the most distressing aspect (for both patient and relatives) and often the reason for hospitalization [17,18]. In the early descriptions of HD, more attention was given to its cognitive features and dementia. Starting in the literature published in the 1990s, many facets of HD have been described including specific psychiatric aspects. Behavioral and psychiatric symptoms (also called prodromal) often precede the manifestation of motor abnormalities of HD [19]. Historical description estimated rates for lifetime prevalence of psychiatric disorders among HD patients vary widely between 33% and 76% [20,21].

Several recent studies have described neuropsychiatric symptoms including depressed mood, mania, irritability, anxiety, apathy, obsessions-compulsions, and psychosis. Prevalence variation depends on the different methods used to detect and evaluate (e.g., interview, rating scales, self-report questionnaires) (Table 1) [1,22,23,24,25].

This result contrasts with incidence rates found in other neurodegenerative diseases (e.g., Parkinson’s disease, in which an incidence rate of major depressive disorder (MDD) of 2% per year is reported) [26,27]. Furthermore, switching to a different psychiatric disorder is not rare. Many factors, including rigid diagnostic criteria, presence of physical symptoms, cognitive impairment, and patient medical history collected by informal caregiver interviews are considered confounding factors [22].

### 3.1. Affective Disorders

The most frequent psychiatric sign occurring in HD patients consists of a depressive symptomatology (DS) [28]. The diagnosis is sometimes difficult because somatic expression of depressed mood (i.e., apathy, inactivity, and weight loss) also occurs in HD patients without psychiatric problems [13]. Estimated prevalence of depression in HD varies widely, ranging from 9% to 63%, with several studies suggesting rates between 40% and 50% (Table 2) [29,30,31,32,33,34,35,36,37,38,39,40]. Kirkwood et al. (2001) suggested that sadness and depression were two of the earliest symptoms at HD onset detected by first-degree relatives’ reports [37].

The development of DS in HD could be a direct result of cerebral degeneration, for which several neuropathological mechanisms have been proposed [41,42] linked to an early neuronal loss in the medial caudate, which has connections to limbic structures [43,44,45]. However, depression might equally well be a consequence of other factors, such as a psychological reaction to being at risk for Huntington’s disease, having experienced an insecure and harmful environment, and/or awareness of the disease’s onset. From a physiological point of view, only a few studies investigated the presynaptic dopaminergic function and no data are yet available on associations with affective symptoms [46,47].

Many studies have found that depressive symptoms precede the onset of motor symptoms but no relation between the occurrence of depressive symptoms and disease duration has so far been reported [48,49]. However, depression may negatively correlate to cognitive decline, which is possibly the result of concurrent decreasing illness awareness [38] and worsened cognitive performance. This is an important point because, if recognized, depression may be properly treated with potential positive consequences on emotional, cognitive, and physical well-being [50]. Paulsen et al. analyzed the relationship between depressive symptoms and disease stage [36] and found the highest rates in the first stages of the disease and lower rates in last stages.

Van Duijn et al. [1,29] described depression as a common characteristic of the disease course, that tends to correlate with the proximity to the clinical onset. Depression seems to increase during the disease course but it is not relevant in the last stage. Furthermore, authors reported that depression is not related to CAG length.

However, in an observational study (the PREDICT protocol study), Epping et al. [30] found that the prevalence of symptoms in prodromal HD patients is not different than the general population. Julien et al. [34] suggested that depression in this early phase cannot be attributed to a general psychological condition linked to being at risk, since it occurs only in a fraction of mutation carriers. No gender effect was found to be significant. This is in contrast with data on the general population, but in other neurodegenerative diseases a similar pattern is observed.

Although depression is the most common specific psychiatric diagnosis in HD, a smaller number of patients become manic, displaying elevated or irritable mood, impulsiveness, hyperactivity, decreased need for sleep, and grandiosity. Some authors have estimated an average rate of mania (with variable definitions of “mania”) of 4.8%, and others have found hypomania and manic episodes in up to 10% of HD patients [51]. Particular attention should be paid to suicide risk. In his original description of the disease, George Huntington stated that there was “a tendency to insanity and suicide”. The suicide rate estimate for HD patients is traditionally 4–6 times higher than in the general population. However, recent studies have confirmed the need to distinguish suicide ideation from attempted suicide, as well as considering differently several groups with different characteristics in order not to introduce confounding data. In particular, depressive symptoms, anxiety, and benzodiazepine consumption are considered risk factors (Table 3) [32,35,52,53,54]. A history of suicide attempts and the presence of depression are strongly predictive of suicidal behavior in prodromal HD. Suicide risk increases in individuals who are closer to motor diagnosis [41].

### 3.2. Anxiety

Anxiety has often been reported in HD patients, independent of gender (17%–61%), both due to the course of the disease and from the neurodegenerative process itself. There are contrasting data regarding the critical stage for anxiety and depression to arise, with some studies identifying stage 2 as the most critical and others suggesting stages 4–5. Moreover, some studies have reported that the stage of illness in HD patients does not influence anxiety levels, contrary to depression symptoms [56]. As stated above, assuming benzodiazepine seems to be an independent risk factor for suicide (Table 4) [22,23,24,34,36,38,52].

### 3.3. Irritability and Aggression

Irritability (Irr) is a common clinical problem in patients with neuropsychiatric disorders. It has been described in HD, traumatic brain injury [57,58], dementias [59], and Parkinson’s disease [60].

Studies have linked irritability and hostility to other aspects of morbidity, including treatment non-adherence [61], suicide attempts [62,63], and violence. While the term “irritability” is widely used in descriptions of patient behavior, but it remains poorly defined. This lack of consensus prompted a definition of Irr, in addition to arguing that irritable mood is separate from other mood disorders such as depression.

Irritation may be defined as a temporary psychological state characterized by impatience, intolerance, and poorly controlled anger. It includes elements of anger, aggression, and reduced impulse control and can occur independently of depression. Prevalence of Irr among HD patients varies between 35% and 73% (Table 5) [23,24,29,38,63,64,65].

Irr without a prior history of similar symptoms occurs in most HD patients and seems to precede motor symptoms in gene carriers [23,66]. Often Irr is described as the first sign of the disease in pre-symptomatic patients with HD [1] but it may occur during all stages of the disease, more frequently in patients whose neurological symptoms have been present for 6 to 11 years [50].

The prevalence of moderate to severe irritability/aggression increased during the disease course [29,67].

Increasing degeneration of the striatum and the orbitofrontal-subcortical circuit contributes to the development of socially inappropriate behaviors that initially may be manifested as subtle irritability and, in late-stage disease, as aggressive behavior [68]. Nevertheless, it is established that some patients may show paradoxical “aggressive” behavior or behavioral disinhibition following benzodiazepine consumption [63]. Taken together, Irr, depression, and anxiety are three salient clinical features of pre-symptomatic HD.

### 3.4. Obsessivity

The presence of obsessive and compulsive symptoms (OCs) has been previously documented in patients with HD as less common than other psychiatric symptoms [65,69,70,71,72]. However, a study by Marder et al. reported that 22.3% had obsessive and compulsive symptoms at their first clinical visit, suggesting that these symptoms may be more common than previously recognized in this population. This symptom shows an increase with disease progression with a trend similar to the one of depression and anxiety in the end stage of the disease. Anderson et al. reported that patients with severe OCs have higher psychiatric co-morbidity (e.g., depression, aggression, and delusions) and neuropsychological deficits.

OCs prevalence varies from 7% to 50% and this association is not surprising, as dysfunction of cortico-striatal connections is also a characteristic of primary disease [67,73,74]. The prevalence of moderate to severe OCs increased progressively [29]. van Duijn et al. also reported a higher than normal prevalence of OCs in preclinical mutation carriers [1].

Many HD gene-carriers show personality changes with mental inflexibility in early stages, possibly signaling future OCs (Table 6) [23,29,33,38,65,75].

### 3.5. Psychosis

Prevalence of psychotic symptoms (PS), including paranoia and delusional and psychotic states resembling various types of schizophrenia-like psychosis, varies between 3% and 11% [1]. The association of PS with HD is a consequence of a relative hyperdopaminergic state (Table 7) [23,24,29,38,40,48,76,77].

Until the first half of the twentieth century, HD patients were often misdiagnosed with dementia praecox or schizophrenia. Now, the low prevalence of PS may be explained by the relatively high use of antipsychotics. PS do not have a specific presentation and must be carefully distinguished from non-organic mental diseases. Some of the patients have a peculiar presentation, as reported by some case reports.

A lack of clinical fluctuation course is typical of a delirium state. PS are usually associated with increasing cognitive impairment and tend to become less overt as the disease progresses. They were found to be particularly important in an advanced stage of the disease. Those patients with an early age at onset of HD seem to be at an increased risk of psychoses [71]. A very low prevalence should be explained by a frequently use of neuroleptics [29].

### 3.6. Apathy and Neuropsychological Deficits

Apathy (AP) has been described both as a symptom (i.e., of mood disorder, altered level of consciousness, or cognitive impairment) and as a syndrome [78,79]. It is defined as a disorder of motivation, with the loss of or diminished goal-directed behavior, cognitive activity, and/or emotion, as well as functional impairments that are attributable to apathy [80,81]. Clinically, it is associated with a decline of daily living activities causing a great burden of disease and distress in caregivers, [82] also after adjusting for the presence of motor and cognitive deficits. Sometimes it is hard to discriminate apathy from depression core symptoms [21]. Prevalence of AP is a common neuropsychiatric feature of HD and varies from 52% to 76% (Table 8) [24,29,38] and, once present, tends to persist or worsen, with highest rate in the late stage of disease [29].

Damage to anterior cingulate-subcortical circuit structures has in particular been related with motivational disorders, including apathy, which may also be the case in HD [49,82]. AP has also been correlated with other symptoms of HD, particularly with cognitive impairments, including problems with working memory, planning, problem solving, and executive function deficits [83].

Psychomotor abilities (e.g., hand-eye coordination tasks such as throwing a ball or typing) are significantly impaired, showing a consistent decline across disease progression, while difficulties in visuospatial and memory abilities occur later in HD [82]. In patients with dementia and apathy, a faster cognitive and functional decline has been found compared to patients without apathy. One final example of frontal behaviors that might be relevant in HD is decreased awareness or loss of insight. Although anecdotal evidence of decreased awareness of symptoms has been reported in HD, few studies have investigated this area. Neuropsychological deficits seem to be particularly relevant among patients with early HD onset.

Many motor and non-motor symptoms in HD have been attributed to subcortical dysfunction (e.g., striatum) and the connecting circuitry with the frontal lobes [84]. For example, executive dysfunction on cognitive tasks (i.e., set shifting, response inhibition) has been linked with the dorsolateral subcortical circuit and dorsolateral prefrontal cortex deficits [85].

On the other hand, neuroleptic sensitivity may also impact apathy, in particular increasing sedation and emotional blunting [86].

### 3.7. Disinhibition, Impulsivity, and Sexual Disorders

Fewer studies have examined disinhibition, which includes impulsivity, hyperactivity, emotional lability and “acting out”, in HD and they have not found this symptom to be particularly prominent in these patients [83]. Changes in sexuality have been well documented in HD. Disorders of sexual functioning, such as hypoactive sexual desire and inhibited orgasm have been reported in men, with rates of 63% and 56%, respectively [87]. In female patients, 75% had hypoactive sexual desire, and 42% had inhibited orgasm. Also, sexual aberrations, such as sexual assault, promiscuity, incest, indecent exposure, and voyeurism, have all been described in HD [64]. The etiology of these abnormalities may be related to specific psychiatric syndromes prevalent in HD, such as mania, to the underlying neurotransmitter deficits of HD, or to medication effects. There is some evidence that male HD patients with both inhibited orgasm and increased sexual interest are at higher risk of developing paraphilias [88].

Hypersexual behavior is more prevalent in men, ranging from 3.9%–30% vs. 2.1%–25% in women [72,87,88]. In a pioneer study, Dewhurst et al. [72] reported in their article some social consequences of these symptoms. In 30 out of 102 patients studied, the authors found abnormal sexual behaviors, with 18.6% showing hypersexuality. In another study, Bolt [88] found that only 20 (6%) out of 334 patients had increased libido or sexual deviation, and Oliver [89] also reported 6% of patients displaying similar behavior.

Craufurd et al. interviewed 134 patients attending an HD management clinic using the Problem Behaviors Assessment for HD (PBA-HD) [36]. Uninhibited sexual behavior was reported by 6% of the patients, while demanding or persistent sexual behavior was described in 5% (Table 9) [38,88].

### 3.8. Pre-Symptomatic HD and Psychiatric Disorders

Previous findings have suggested, although inconclusively, that psychopathology as well as cognitive dysfunction may precede the onset of motor symptoms in many patients [90,91]. Now, with the availability of a genetic test, it is possible to identify individuals who have CAG trinucleotide repeat expansion in the pathological range but are not yet showing sufficient motor signs for the diagnosis of clinical disease (i.e., pre-symptomatic gene carriers/subjects). Thus, it is now possible to study the relationship between genetic data and individuals’ characteristics before disease onset. Indeed, several studies have found subtle motor, cognitive, psychiatric, and neuroimaging abnormalities in HD years before diagnosis [92].

Regarding depressive symptoms, data on pre-symptomatic patients are still controversial, with some studies reporting a greater prevalence of MDD and depressive symptoms in both symptomatic and pre-symptomatic mutation carriers than non-carriers, and others reporting no differences [1,93]. From the existing literature, it is unclear how early behavioral manifestations of frontal dysfunction occur in HD. Duff et al. [19], using a pre-HD cohort, have previously reported on subtly elevated psychiatric symptoms in individuals prior to diagnosis. Some of these symptoms may reflect frontal disturbances (e.g., obsessive-compulsiveness, interpersonal sensitivity, hostility) in pre-HD. The authors hypothesized that pre-HD, expansion-positive individuals would report higher levels of frontal behaviors compared to expansion-negative individuals. Not only are apathy, disinhibition, and executive dysfunction more prevalent in the gene-expanded group, they are also associated with other markers of disease progression in HD.

## 4. Conclusions

Psychiatric disturbances are a constituent core of HD; this is known by the disease’s first description. The pathways leading to psychopathology in HD are still not completely clear. Indeed, several findings suggest that both neuropathology and environmental stress contribute to the occurrence of neuropsychiatric phenomena [15]. One characteristic feature is continuous change in the clinical presentation of symptoms, complicating nosography and pharmacological treatment.

It is clear that not only depression and anxiety are typical symptoms of HD, but also a number of other psychological, behavioral, and cognitive symptoms that may deeply impair quality of life of patients and relatives. Diagnosing and acknowledging the presence of psychopathology in HD is of major importance and may help patients and their families cope better with the severe symptoms of this progressive disease. It is thus vital to confront these problems from the beginning of the disease journey. First of all, diagnosis communication should be the first step of a long trip in which patients and relatives must know what is going to happen and how to face it [17]. Communication support and psychological counseling should then be guided by the knowledge of the psychiatric course that the disease could take. At the same time, a good communication channel should always be open to patients, relatives, and the medical team in order to enable timely response to the rise of psychiatric or cognitive symptoms. Pharmacological treatment should be properly used in a timely fashion (e.g., avoiding Xenazine in patients with severe depression at high risk of suicidality and preferring anti-impulsive treatment) in order to prevent breakdown and potential social and familial consequences. In addition, drug interactions should be also monitored to prevent adverse or even paradoxical reactions. An adequate symptomatic treatment can improve the quality of life of HD patients and their caregivers [94].

The HD treatment (psychopharmacological and/or psychological support) is based at present on a difficult integration of the still scarce experimental evidence and experience gained in the field, integration that is left to the clinician (or the clinical team), with indisputable and urgent decision-loading. In an attempt to define a good clinical practice in the context of HD, some treatment algorithms have been developed whose initial assumption remains the need for tailored interventions, responding not only to the patient’s overall health condition (co-morbidity and contraindications to treatment) but also to the patient’s needs and preferences.

## Figures and Tables

**Table 1:** Neuropsychiatric disturbances in mutation carriers.

Authors	Number of Patients	Study Design	Incidence or Prevalence
Reedeker W. et al. [22]	91	Two-year observational cohort study in symptomatic and pre-symptomatic patients	14 out of 91 patients had a psychiatric disorder within two years after symptoms onset or genetic diagnosis (15%).
van Duijn E. et al. [1]	154	Two-year observational cohort study in symptomatic and pre-symptomatic patients	36 out of 140 patients had a psychiatric disorder (25.7%). Symptomatic mutation carriers did not differ from presymptomatic mutation carriers.
Murgod U. A. et al. [23]	26	One-year prospective cohort study in symptomatic and pre-symptomatic patients	Three out of 26 of patients (11.5%) had psychiatric symptoms.
Paulsen J. S. et al. [24]	52	Cross-sectional observational study in symptomatic and pre-symptomatic patients	98% of patients had at least one neuropsychiatric symptom.
Leroi I. et al. [25]	21	Cross-sectional observational study in symptomatic patients	17 out of 21 patients (81%) showed psychiatric disorders.

**Table 2:** Depression in mutations carriers.

Author	Number of Patients	Study Design	Incidence/Prevalence
Van Duijn et al. [29]	1993	Observational cohort study in pre-symptomatic and symptomatic patients	586 patients (29.4%) showed middle depression; 254 (12.7%) moderate to severe.
Epping [30]	3803	Observational cohort study in pre-symptomatic patients	Depressive symptoms were frequent (313 out 803 had symptoms of depression, ranging from minimal to severe), but did not increase as a function to the proximity of diagnosis.
Thompson J. C. et al. [31]	111	Three-year observational cohort study in manifest HD patients	Depression symptoms, with longitudinal prevalence rates from 18% to 71%.
Wetzel H. H. et al. [32]	1941	Retrospective multi-site study on symptomatic and pre-symptomatic patients	48.2% sought help for depression and 40.3% were prescribed anti-depressant medication.
Anderson K. E. et al. [33]	1642	Observational cross-sectional study in symptomatic patients	801 patients (48.8%) reported a hystory of treatment for depression.
Julien C. L. et al. [34]	204	Case-control study on symptomatic, pre-symptomatic and non-carriers participants	18 mutation carriers out 89 had a history of major depression.
Marshall J. et al. [35]	254	Case-control study on symptomatic, pre-symptomatic and non-carriers participants	Preclinical mutation carriers with motor abnormalities and HD patients showed higher level of depression than other groups.
Paulsen J. S. et al. [36]	2835	Observational cohort study in manifest HD patients	50.3% of participants reported seeking treatment for depression. Lower levels of depression were found in later stages of the disease.
Leroi I. et al. [25]	21	Case-control study comparing HD manifest patients, Degenerative Cerebellar (DC) disease, and healthy controls	10 out 21 (42.8%) HD patients reported mood disorders. Depression was significantly higher in HD and DC patients than healthy controls.
Kirkwood S. C. et al. [37]	1238	Observational cohort study in HD manifest patients	Prevalence of depression varied from 7.5% to 22.5% at different disease stages (279, <1 year; 289, 2–5 years; 180, 6–10 years; 93, >10 years) with the lowest rate at the end of the illness.
Murgod U. A. et al. [23]	26	One-year prospective cohort study in symptomatic patients	Depression was extremely common and was present in 17 out of 26 patients (65.4%).
Craufurd D. et al. [38]	134	Observational cross-sectional study in HD manifest patients	33% of patients reported depression symptoms.
Kulisevsky J. et al. [39]	29	Case-control study comparing HD patients and other movement disorders	HD patients had higher scores of depression on the Neuropsychiatric Inventory evaluation than patients with a hypokinetic movement disorder.
Pflanz S. [40]	86	Retrospective study, in manifest HD patients (not every patient had HTT test)	42 out of 86 patients (52%) reported clinical depression.

**Table 3:** Suicidality in mutation carriers.

Author	Number of Patients	Study Design	Incidence/Prevalence
Hubers A. A. M. et al. [52]	2106	Observational prospective multicentric study on symptomatic and pre-symptomatic mutation carriers	163 symptomatic and six pre-symptomatic patients (169 out of 2016, 8%) had suicidal ideation at baseline. At follow-up, among the 945 patients without suicidal ideation at baseline, 52 had developed it (6%).
Hubers A. A. M. et al. [53]	152	Two-year observational cohort study on symptomatic and pre-symptomatic	31 out of 152 patients (20%) reported suicidality at baseline, without differences between symptomatic and pre-symptomatic. At follow-up, seven out of 100 (7%) developed suicide ideation.
Wetzel H. H. et al. [32]	1941	Retrospective multi-site study on symptomatic and pre-symptomatic patients	360 out of 1941 reported current suicidal ideation, while 26.5% admitted a previous history of suicidal ideation. 184 patients (9.5%) reported at least one suicide attempt.
Fiedorowicz J. G. et al. [54]	735	Retrospective study on pre-symptomatic patients	12 out of 735 (1.6%) attempted suicide, while one completed suicide.
Paulsen J. S. et al. [55]	4171	Observational study on pre-symptomatic and symptomatic patients and people at risk for disease	Suicidal ideation increased from no and soft signs of disease to possible HD (up to 23.5%), while decreasing in manifest HD patients.

**Table 4:** Anxiety in mutations carriers.

Authors	Number of Patients	Study Design	Incidence/Prevalence
Hubers A. A. M. et al. [52]	2106	Observational prospective multicentric study on symptomatic and pre-symptomatic mutation carriers	641 of 1937 (33.1%) reported significant anxiety levels without suicidal ideation; 114 of 69 (67.5%) with suicidal ideation had also high anxiety.
Reedeker R. C. et al. [22]	106	Two-year observation cohort study on pre-symptomatic, symptomatic patients and a non-carrier control group	Three patients had a diagnosis of anxiety at baseline and four were diagnosed after two years.
Julien C. L. et al. [34]	204	Case-control study on symptomatic, pre-symptomatic and non-carriers participants	No differences in anxiety levels were found between groups.
Paulsen J. S. et al. [36]	2835	Observational cohort study in patients with manifest HD	41% endorsed symptoms of anxiety.
Paulsen J. S. et al. [24]	52	Observational cross-sectional study in patient with manifest HD	51.9% were found to have anxiety symptoms.
Murgod U. A. [23]	26	One-year prospective study in symptomatic patients	61.5% showed anxiety symptoms.
Craufurd D. et al. [38]	134	Observational cross-sectional study in HD manifest patients	37% had significant anxiety levels.

**Table 5:** Irritability in mutation carriers.

Authors	Number of Patients	Study Design	Incidence/Prevalence
Van Duijn E. et al. [29]	1993	Observational cohort study in pre-symptomatic and symptomatic patients	493 patients (24.7%) showed mild irritability; 277 showed (13.9%) moderate to severe irritability.
Reedeker N. et al. [64]	130	Two-year observation cohort study on pre-symptomatic, symptomatic patients and a non-carriers control group	45 out of 130 (35%) had high irritability scores. Carriers had significantly higher scores than non-carriers.
Orth M. et al. [65]	1766	Observational cohort study on patients with manifest HD	244 of 1343 patients (19.1%) reported disruptive or aggressive behavior.
Craufurd D. et al. [38]	134	Observational cross-sectional study in HD manifest patients	44% reported irritability severity score of 2 or more.
Murgod U. A. et al. [23]	26	One-year prospective study in symptomatic patients	19 out of 26 patients (73%) reported an irritable behavior.
Paulsen J. S. et al. [24]	52	Observational study in patient with manifest HD	65.4% of patients was reported to show irritability.
Marder K. et al. [66]	960	Prospective observational study on patients with manifest HD	63.6% showed some aggressive behavior at their first visit.

**Table 6:** OCs in mutation carriers.

Authors	Number of Patients	Study Design	Incidence/Prevalence
van Duijn E. et al. [29]	1993	Observational cohort study in pre-symptomatic and symptomatic patients	252 out of 1993 patients (12.6%) showed mild OCs; 363 showed (13.2%) moderate to severe OCs.
Beglinger L. et al. [76]	3964	Obervational cohort study on individuals at risk for HD	OC symptoms increased with greater diagnostic certainty and functional impairment. High rates of obsessive (13%) and compulsive (7%) behaviors were found also in patients with soft neurological signs.
Craufurd D. et al. [38]	134	Observational cross-sectional study in HD manifest patients	5% reported obsession symptoms.
Murgod U. A. et al. [23]	26	One-year prospective study in symptomatic patients	Four out of 26 patients reported obsessions and three reported compulsions.
Anderson K. E. et al. [33]	1642	Observational study in symptomatic patient	446 out of 1642 patients (27.2%) had OC symptoms; 217 (48.7%) reported only obsessive symptoms, 165 (39.6%) experienced both obsessive and compulsive symptoms, and 52 (11.7%) had only compulsions.
Marder K. S. et al. [66]	960	Prospective observational study on patients with manifest HD	22.3% had obsessive and compulsive symptoms at their first clinic visit.

**Table 7:** Psychosis in mutation carriers.

Authors	Number of Patients	Study Design	Incidence/Prevalence
van Duijn E. et al. [29]	1993	Observational prospective study on presymptomatic and symptomatic patients	58 (2.9%) "mild psychosis“, and 23 (1.2%) scoring "moderate to severe psychosis“. The highest prevalence of psychosis (2.5%) was in stage 3.
Grabski B. et al. [77]	1	Case report	Schizophrenia-like psychotic symptoms in a patient with confirmed Huntington’s disease: a case report.
Chuo Y. P. et al. [78]	1	Case report	Juvenile Huntington’s disease presenting as difficult-to-treat seizure and the first episode of psychosis.
Pflanz S. et al. [40]	86	Retrospective study, symptomatic patients	Prevalence between 8.8% to 11.5%.
Folstein et al. [48]	34	Observational study HD manifest patients and their offsprings	Two out of 34 patients showed signs of a psychotic disorder.
Craufurd D. et al. [38]	134	Observational cross-sectional study in HD manifest patients	5% had signs of a psychotic disorder.
Murgod U. A. et al. [23]	26	One-year prospective study in symptomatric patients	11.5% had signs of a psychotic disorder.
Paulsen J. S. et al. [24]	52	Observational cross-sectional study in motor symptomatic patients	12% was diagnosed with a psychotic disorder

**Table 8:** Apathy symptoms in mutation carriers.

Authors	Number of Patients	Study Design	Incidence/Prevalence
van Duijn E. et al. [29]	1993	Observational prospective study on presymptomatic and symptomatic patients	385 (19.3%) presented mild apathy; 560 (28.1%) presented moderate to severe apathy.
Craufurd D. et al. [38]	134	Cross-sectional study in HD manifest patients	70% showed apathy symptoms. Apathy was highly correlated with duration of illness.
Paulsen J. S. et al. [24]	52	Cross-sectional study HD manifest patients	55.8% presented apathy.

**Table 9:** Sexual dysfunction in mutation carriers.

Authors	Number of Patients	Study Design	Incidence/Prevalence
Craufurd D. et al. [38]	134	Observational cross-sectional study in HD manifest patients	Eight out 134 patients (6%), hypersexuality; 82 (61%) hyposexuality.
Fedoroff J. P. et al. [88]	39	Observational cross-sectional study on HD manifest patients	32 out of 39 patients experienced at least one sexual disorder
